# Synergistic therapeutic strategies for metabolic dysfunction-associated steatohepatitis and type 2 diabetes mellitus: molecular insights and clinical advances

**DOI:** 10.3389/fendo.2025.1753393

**Published:** 2026-01-19

**Authors:** Bo Zhu

**Affiliations:** Vascular Biology Program, Boston Children’s Hospital, Department of Surgery, Harvard Medical School, Boston, MA, United States

**Keywords:** fibrosis, inflammation, insulin resistance, liver-pancreas axis, MASH, pharmacological therapy, type 2 diabetes mellitus

## Abstract

Metabolic dysfunction-associated steatohepatitis (MASH) and type 2 diabetes mellitus (T2DM) are closely linked conditions that share common disturbances in metabolism, inflammation, and fibrotic processes. MASH is characterized by fat accumulation in the liver, hepatocyte damage, and progressive fibrosis, whereas T2DM involves insulin resistance and impaired beta-cell function. The coexistence of these disorders creates a liver and pancreas feedback loop, in which impaired hepatic insulin signaling worsens blood glucose control and high glucose levels further damage the liver. Key cellular contributors include hepatocytes, Kupffer cells, hepatic stellate cells, and pancreatic β-cells, while non-coding RNAs influence lipid metabolism and inflammation. Emerging therapies, including GLP1 receptor agonists, dual incretin agents, PPAR modulators, thyroid hormone receptor beta modulators, FXR agonists, and FGF analogues, along with lifestyle interventions, show promise in improving both liver and metabolic outcomes. Precision medicine approaches may further refine individualized treatment strategies.

## 1 Introduction

Metabolic dysfunction-associated steatohepatitis (MASH), previously termed non-alcoholic steatohepatitis, has become the most common cause of chronic liver disease worldwide, mirroring the rise in obesity and type 2 diabetes mellitus (T2DM) (1–3). Current epidemiological data suggest that MASH affects roughly 3-5% of the general population in Western countries, with markedly higher prevalence among individuals with obesity, insulin resistance, or T2DM (4–6). Without intervention, MASH can progress to advanced fibrosis, cirrhosis, hepatic decompensation, and hepatocellular carcinoma, contributing substantially to global morbidity and mortality (7). T2DM, affecting more than 500 million people globally, accounts for nearly all cases of diabetes (8). Both disorders arise from shared disturbances in insulin signaling, lipid handling, and chronic low-grade inflammation, indicating a convergent metabolic origin that facilitates mutual disease progression (9, 10).

**Figure 1:**
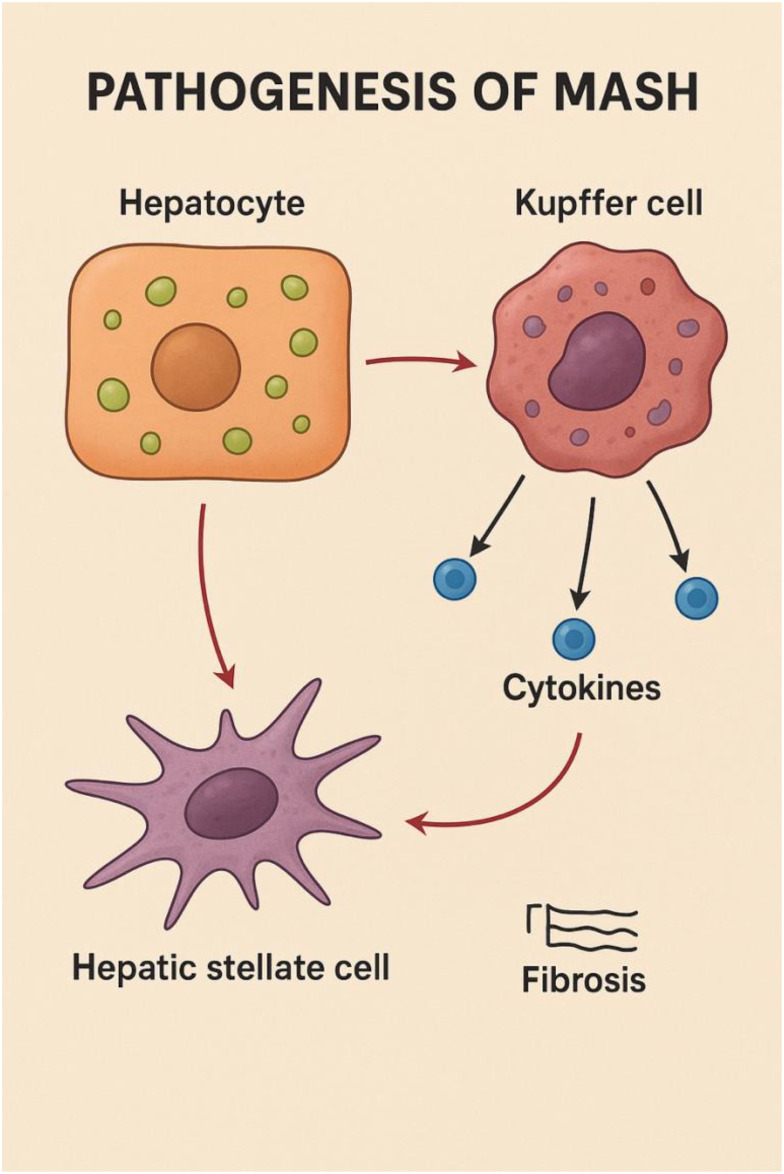
Pathogenesis of metabolic dysfunction–associated steatohepatitis (MASH). The figure illustrates key cellular interactions driving the progression of MASH. Lipid-laden hepatocytes release signals that activate liver-resident macrophages (Kupffer cells). Activated Kupffer cells secrete pro-inflammatory cytokines, which further amplify hepatic inflammation and contribute to hepatocellular injury. These cytokines, together with hepatocyte-derived stress signals, stimulate hepatic stellate cells, promoting their transition into a myofibroblast-like phenotype. Activated stellate cells deposit extracellular matrix components, leading to fibrosis and progressive liver remodeling characteristic of MASH (Created with BioRender.com).

At the mechanistic level, MASH develops through coordinated dysfunction among hepatocytes, Kupffer cells, and hepatic stellate cells (HSCs) (**Figure 1**). Excess lipid loading in hepatocytes induces lipotoxic stress, triggering cell injury and apoptotic signaling. Injured hepatocytes release damage-associated molecular patterns (DAMPs), which activate Kupffer cells. Activated Kupffer cells secrete pro-inflammatory mediators, including tumor necrosis factor-α (TNF-α), interleukin-1β (IL-1β), and interleukin-6 (IL-6), which amplify hepatic inflammation and stimulate HSCs (11). Persistent activation of HSCs drives extracellular matrix deposition and progressive fibrosis, facilitating the transition from simple steatosis to steatohepatitis and ultimately cirrhosis (1).

**Figure 2:**
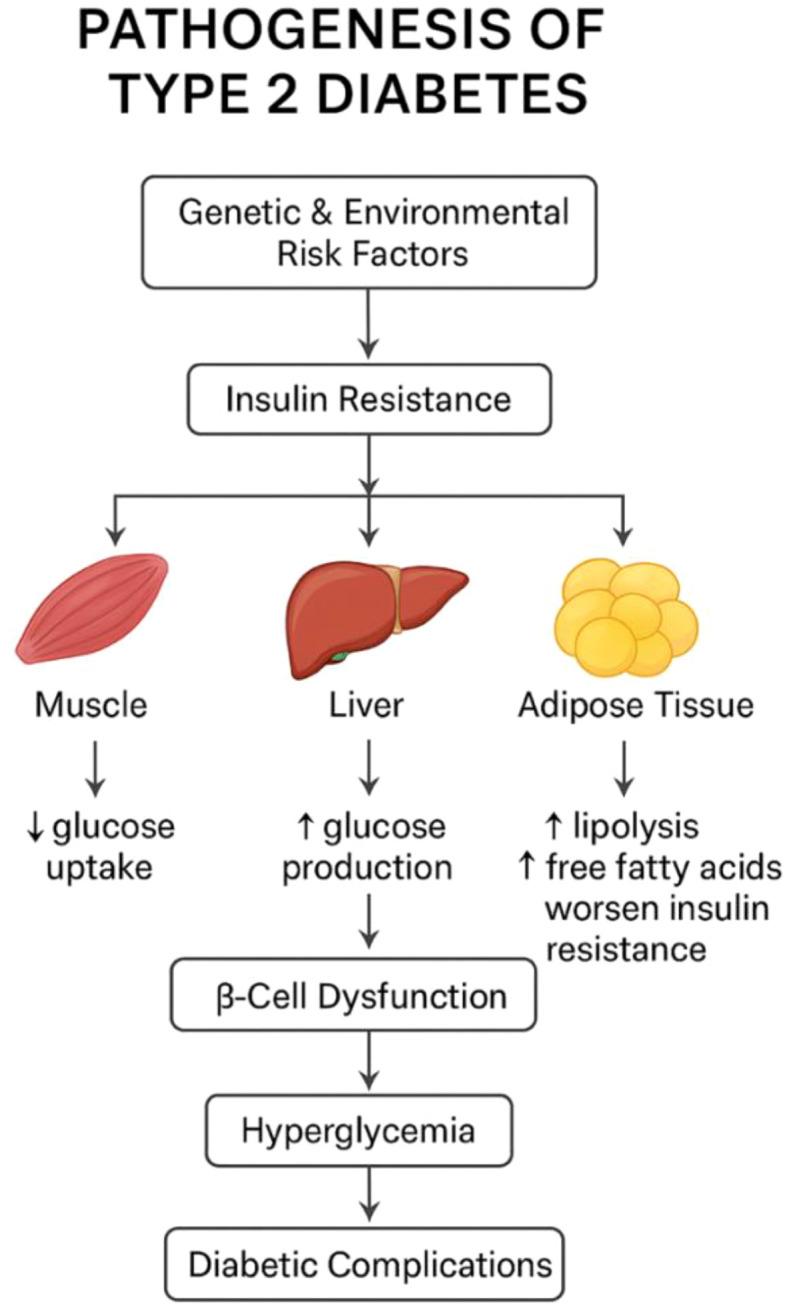
Pathogenesis of type 2 diabetes. Schematic overview of the major contributors to type 2 diabetes development. Genetic and environmental factors promote insulin resistance, which affects skeletal muscle, liver, and adipose tissue. In skeletal muscle, insulin resistance reduces glucose uptake. In the liver, it increases hepatic glucose production. In adipose tissue, enhanced lipolysis elevates circulating free fatty acids, further aggravating insulin resistance. Progressive β-cell dysfunction leads to hyperglycemia and subsequent diabetic complications (Created with BioRender.com).

**Figure 3:**
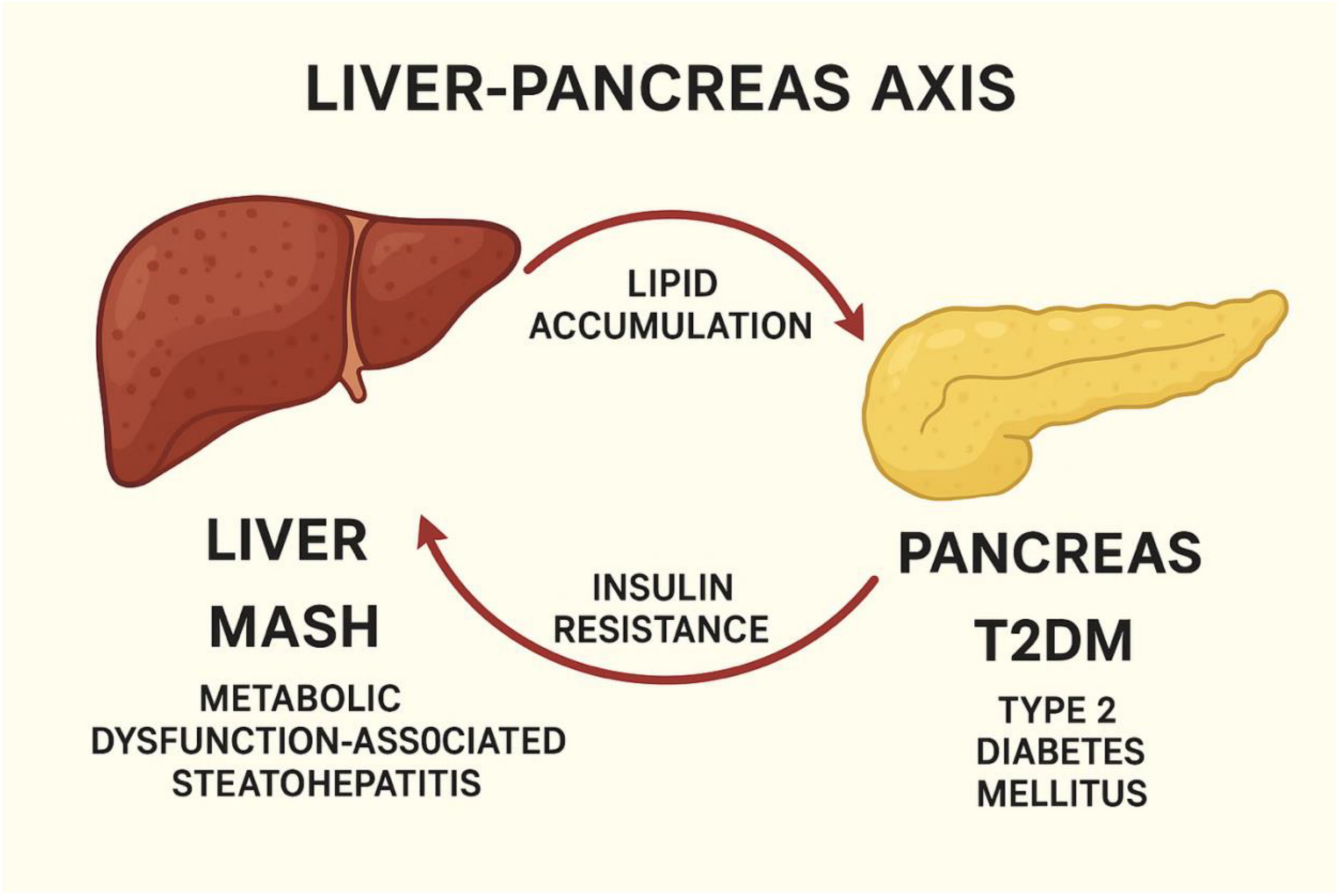
Liver-pancreas axis in metabolic dysfunction. Diagram illustrating the bidirectional relationship between the liver and pancreas in metabolic disease. Lipid accumulation in the pancreas is shown as a downstream effect of liver metabolic dysfunction-associated steatohepatitis (MASH). Increased insulin resistance originating from pancreatic dysfunction contributes to progression of hepatic steatosis and inflammation. Together, these processes create a reciprocal cycle linking MASH and type 2 diabetes mellitus (T2DM) (Created with BioRender.com).

T2DM is defined by systemic insulin resistance and chronic hyperglycemia, both of which are closely intertwined with hepatic lipid accumulation (**Figure 2**). In hepatocytes, impaired insulin signaling disrupts glycogen storage and lipid metabolism, promoting steatosis and worsening hyperglycemia. Compensatory hyperinsulinemia initially maintains glucose control but ultimately contributes to β-cell overload and functional decline (12). The coexistence of MASH and T2DM creates a reinforcing metabolic loop: hepatic insulin resistance accelerates β-cell dysfunction, whereas hyperglycemia and systemic inflammation intensify hepatic injury (13). This reciprocal relationship, often conceptualized as the liver-pancreas axis, represents a central regulatory hub in metabolic homeostasis and an emerging focus for therapeutic innovation (**Figure 3**).

Given the growing global burden of MASH and T2DM and their strong bidirectional interactions, there is an urgent need for therapeutic strategies that address both conditions concurrently. Agents that improve insulin sensitivity, diminish hepatic lipid accumulation, and attenuate inflammatory signaling show particular promise (14). Glucagon-like peptide-1 (GLP-1) receptor agonists, peroxisome proliferator-activated receptor (PPAR) agonists, thyroid hormone receptor-β agonists, fibroblast growth factor 21 (FGF21) analogues, and farnesoid X receptor (FXR) agonists have demonstrated beneficial effects in both preclinical models and clinical studies (15–18). Alongside pharmacotherapy, lifestyle interventions, especially dietary changes, weight reduction, and regular physical activity, remain essential components of long-term disease management and improvement of hepatic outcomes (19).

This review synthesizes current knowledge of the shared molecular pathways linking MASH and T2DM, drawing from mechanistic studies, experimental models, and clinical evidence. We highlight key cellular targets within hepatocytes, Kupffer cells, HSCs, and pancreatic β-cells; evaluate therapeutic agents with dual metabolic and hepatic efficacy; and outline future directions for combination approaches aimed at correcting both metabolic and liver dysfunction. By integrating mechanistic and translational perspectives, this work aims to support the development of next-generation therapies to mitigate the growing dual burden of MASH and T2DM.

## 2 Shared genetic predisposition of MASH and T2DM

**Figure 4:**
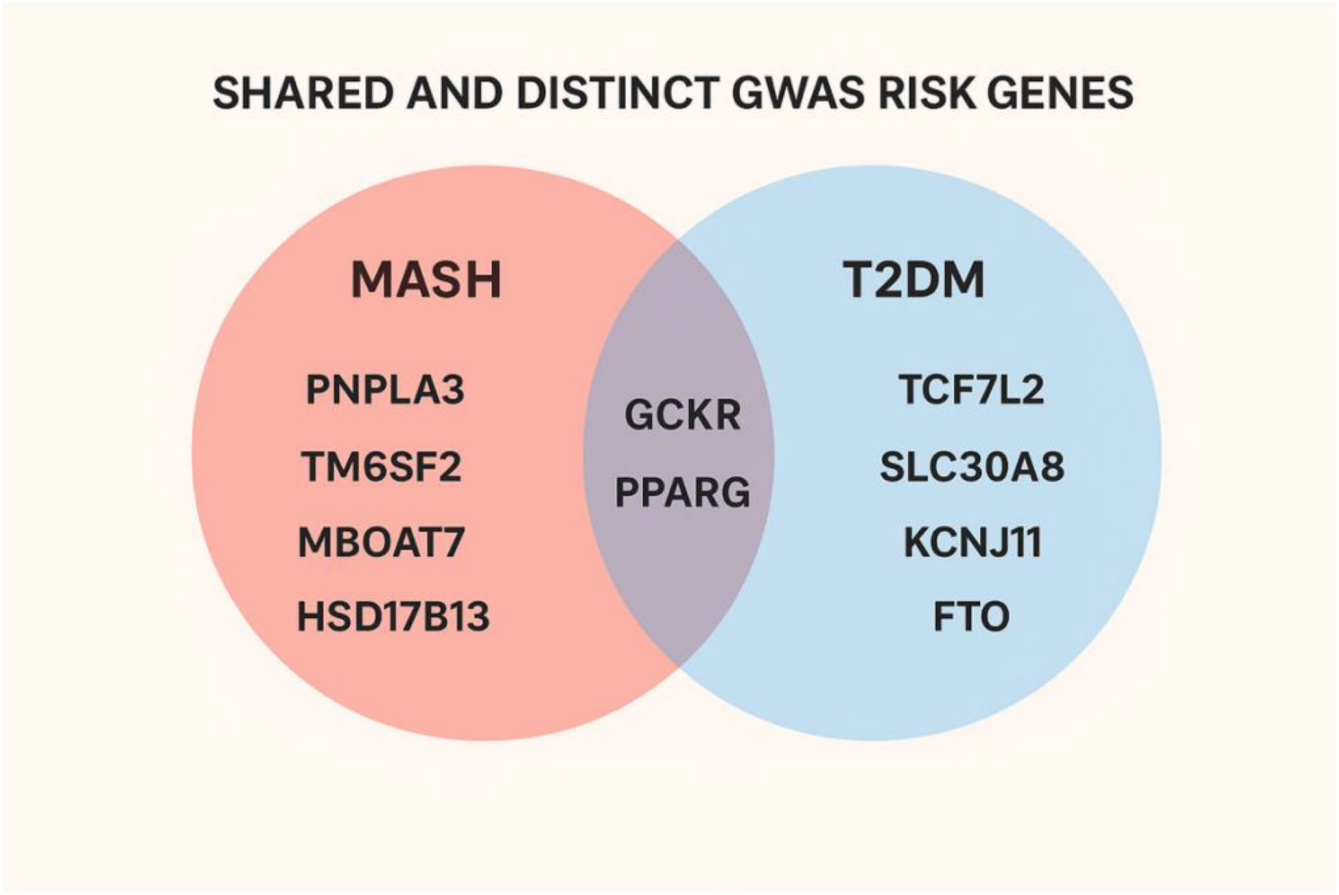
Shared and distinct GWAS-identified genetic risk loci associated with MASH and T2DM. **T**he Venn diagram illustrates representative genetic variants identified through genome-wide association studies (GWAS) that contribute to the pathogenesis of Metabolic Dysfunction-Associated Steatohepatitis (MASH) and Type 2 Diabetes Mellitus (T2DM). MASH-specific risk genes (*PNPLA3, TM6SF2, MBOAT7*, and *HSD17B13*) are shown in the left circle, while T2DM-specific genes (*TCF7L2, SLC30A8, KCNJ11*, and *FTO*) appear in the right circle. Shared genes (*GCKR* and *PPARG*) occupy the overlapping region, reflecting their involvement in both hepatic metabolic dysfunction and systemic insulin resistance. Together, these loci highlight converging and diverging genetic mechanisms underlying both metabolic diseases (Created with BioRender.com).

MASH and T2DM exhibit a significant overlap in genetic susceptibility, primarily through shared pathways such as insulin resistance and lipid metabolism (10). Genetic variants affecting beta-cell function, insulin sensitivity, adiposity, and hepatic lipid handling have been implicated in both disorders (20, 21). Insulin resistance represents a central shared mechanism, with specific genetic variations enhancing vulnerability to both MASH and T2DM (22). Furthermore, inherited susceptibility to obesity, a major risk factor, contributes to the onset of both disorders (23). According to the GWAS Catalog, 333 risk alleles are associated with MAFLD/MASH, while 8,794 are linked to T2DM, with 182 genes implicated in both diseases. Representative risk genes identified in GWAS studies and involved in the pathogenesis of MASH and T2DM are summarized in **Figure 4**.

For MASH, variants in PNPLA3, TM6SF2, GCKR, MBOAT7, and HSD17B13 are particularly influential. PNPLA3 (rs738409, I148M) represents the strongest genetic signal for MAFLD/MASH, promoting hepatic lipid accumulation and inflammation (24). TM6SF2 (rs58542926, E167K) affects hepatic triglyceride content and VLDL secretion, contributing to disease susceptibility (25). GCKR (rs1260326) influences triglyceride levels, VLDL production, and glucose regulation, linking it to both MAFLD and T2DM (26). MBOAT7 (rs641738) increases hepatic fat deposition and fibrosis risk, playing a role in MAFLD/MASH progression (27). Conversely, the HSD17B13 loss-of-function variant (rs72613567: TA) is associated with reduced risk of chronic liver disease and decreased progression from steatosis to steatohepatitis (28).

For T2DM, risk is elevated by variants in TCF7L2, PPARG, SLC30A8, KCNJ11, and FTO. The TCF7L2 variant (rs7903146) influences blood glucose regulation and significantly increases T2DM risk (29). PPARG (rs1801282) affects insulin sensitivity and hepatic lipid metabolism, linking it to both T2DM and MASH (30). SLC30A8 (rs13266634) encodes a β-cell zinc transporter, and its variant elevates T2DM susceptibility (31). KCNJ11 (rs5219, E23K) impacts insulin secretion, further increasing T2DM risk (32). Finally, the FTO variant (rs9939609) contributes to obesity and heightens the risk of T2DM (33).

## 3 Shared pathophysiology of MASH and T2DM

**Figure 5:**
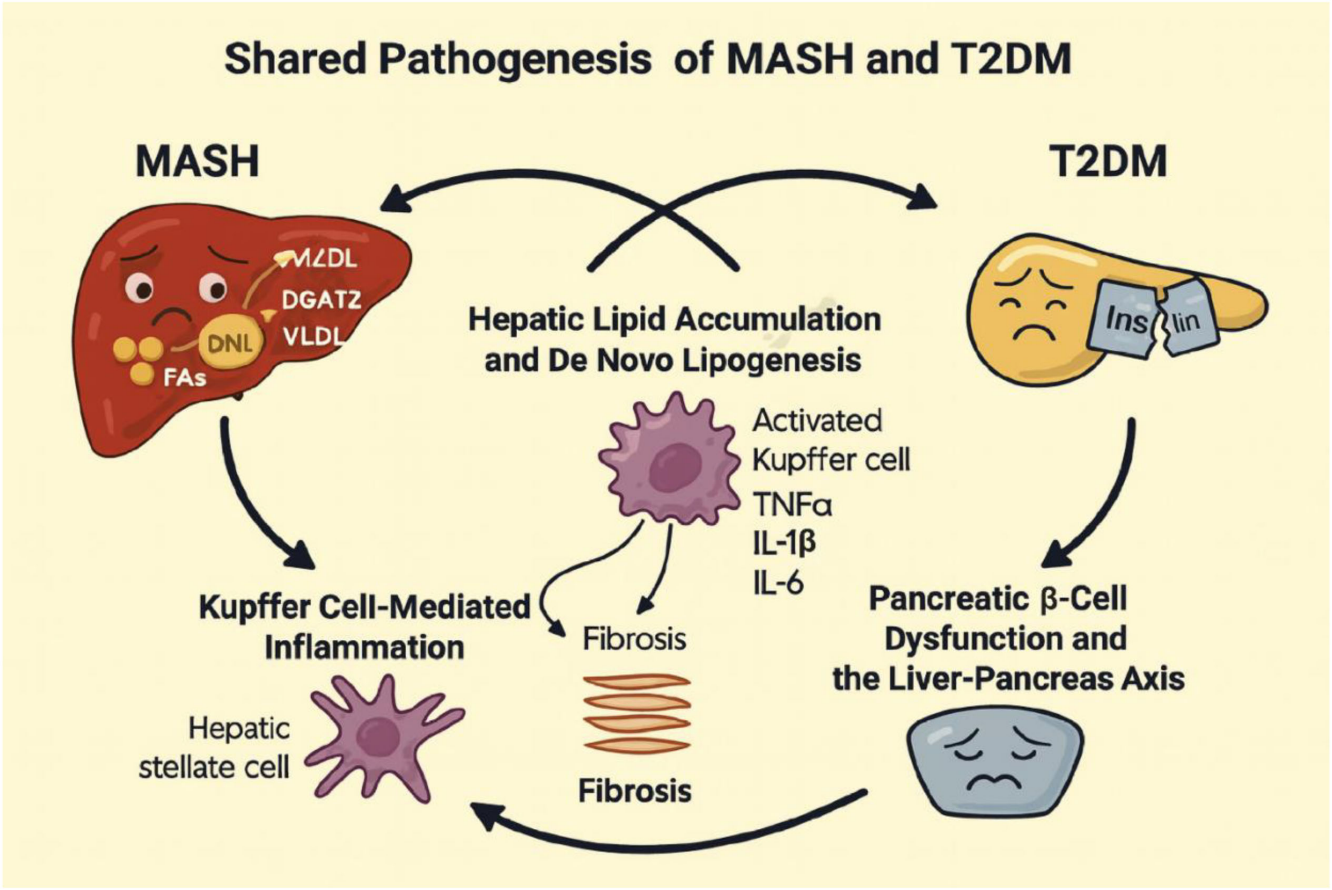
Shared pathophysiological mechanisms linking MASH and T2DM. The diagram illustrates the major convergent pathways underlying both metabolic dysfunction–associated steatohepatitis (MASH) and type 2 diabetes mellitus (T2DM). Excess hepatic lipid accumulation driven by increased *de novo* lipogenesis contributes to steatosis and hepatocellular stress. Lipotoxic injury activates Kupffer cells, promoting release of pro-inflammatory cytokines and recruitment of additional immune cells. These signals stimulate hepatic stellate cell activation and extracellular matrix deposition, leading to fibrosis. In parallel, hepatic insulin resistance and inflammation impair the liver–pancreas axis, driving β-cell stress, reduced insulin secretory capacity, and worsening systemic metabolic dysfunction. Together, these interconnected processes form a self-reinforcing cycle that underlies the shared pathogenesis of MASH and T2DM (Created with BioRender.com).

MASH and T2DM are tightly linked metabolic disorders characterized by convergent pathogenic processes, including insulin resistance, dysregulated lipid handling, persistent inflammation, and fibrotic remodeling (**Figure 5**). Recognition of these shared mechanisms forms the basis for developing therapeutic approaches capable of targeting both conditions simultaneously (10).

### 3.1 Hepatic lipid accumulation and *De Novo* lipogenesis

Hepatic steatosis, a defining feature of MASH, arises from an imbalance between lipid influx, which is driven by *de novo* lipogenesis (DNL), uptake of circulating fatty acids, and adipose tissue lipolysis, and lipid disposal through β-oxidation and very-low-density lipoprotein (VLDL) export (34). DNL is controlled by key enzymatic regulators, including ATP citrate lyase (ACLY), acetyl-CoA carboxylase isoforms ACC1 and ACC2, fatty acid synthase (FASN), and diacylglycerol O-acyltransferase 2 (DGAT2) (35). Findings from hepatocyte-specific knockout models demonstrate that suppressing these enzymes reduces steatosis and attenuates fibrotic progression. For instance, ACLY deletion lowers cytosolic acetyl-CoA, thereby limiting fatty acid synthesis (36); ACC1/2 inhibition decreases malonyl-CoA availability for FASN-mediated chain elongation (37); and DGAT2 blockade restricts triglyceride formation, preventing lipid droplet accumulation and reducing hepatocellular lipotoxicity (38). Additionally, hepatic follistatin (FST) plays a protective role against excessive lipid accumulation in the liver by inhibiting of lipid synthesis and promoting energy expenditure (39, 40).

In T2DM, hepatic insulin resistance aberrantly activates DNL even in the context of hyperglycemia, thereby worsening steatosis and perpetuating a metabolic feedback loop (41). Insulin-resistant hepatocytes contribute to compensatory hyperinsulinemia, increasing β-cell burden and destabilizing glycemic control (8). Thus, therapeutic strategies that modulate hepatic lipid metabolism hold promise for improving both MASH pathology and systemic glucose homeostasis.

### 3.2 Kupffer cell-mediated inflammation

Chronic inflammation is a defining component of both MASH and T2DM, with liver-resident macrophages (Kupffer cells) serving as key drivers of hepatic injury (42). Under conditions of hepatocyte stress or lipotoxicity, Kupffer cells are activated by damage-associated molecular patterns (DAMPs) and pathogen-associated molecular patterns (PAMPs). Once activated, they release pro-inflammatory cytokines such as tumor necrosis factor-α (TNF-α), interleukin-1β (IL-1β), and interleukin-6 (IL-6), along with chemokines that recruit circulating monocytes to the liver. These mediators amplify hepatocellular damage and stimulate hepatic stellate cells (HSCs), thereby initiating and propagating fibrogenesis (43).

Kupffer cell-dependent signaling pathways further intensify this inflammatory cascade. Activation of the NLRP3 inflammasome promotes the maturation of IL-1β, while Toll-like receptor 4 (TLR4) senses lipopolysaccharide (LPS) and endogenous ligands released during hepatocyte injury, sustaining inflammatory activation (44). Transcriptional regulators such as CCAAT/enhancer-binding protein-β (C/EBPβ) and Yes-associated protein (YAP) modulate cytokine expression programs and contribute to disease progression (45).

In T2DM, systemic insulin resistance fosters a chronic pro-inflammatory state (46). Obesity-associated adipose tissue expansion increases macrophage infiltration and cytokine secretion, which exacerbates hepatic inflammation. Circulating free fatty acids can further activate TLR4 on Kupffer cells, linking peripheral metabolic dysfunction to liver injury (47). This shared inflammatory foundation highlights the importance of targeting innate immune pathways when developing therapies that address both MASH and T2DM.

### 3.3 Hepatic stellate cell activation and fibrosis

Hepatic stellate cells (HSCs) are the primary fibrogenic effector cells in the liver (48). In their quiescent state, HSCs store vitamin A and contribute to extracellular matrix (ECM) homeostasis. Upon exposure to cytokines, chemokines, or oxidative stress, they transition into activated, myofibroblast-like cells that produce collagen types I and III, fibronectin, and additional ECM components, ultimately driving fibrotic remodeling (49).

Multiple signaling pathways orchestrate HSC activation, including transforming growth factor-β (TGF-β)/SMAD, Hippo/YAP–TEAD, retinoid X receptor-α (RXRα), activating transcription factor 4 (ATF4), and SERPINE1. TGF-β stimulates its receptor complex to initiate SMAD2/3-dependent transcription of pro-fibrotic genes (50). YAP, a central regulator of mechanotransduction, promotes HSC proliferation and matrix deposition in response to increased tissue stiffness (51). RXRα and ATF4 modulate lipid metabolism and cellular stress signaling, thereby influencing the activation state of HSCs (52, 53). In parallel, SERPINE1 impairs ECM degradation, reinforcing the fibrotic response (54).

In T2DM, hyperglycemia and insulin resistance enhance oxidative stress and accelerate the generation of advanced glycation end-products (AGEs), both of which further stimulate HSC activation (55). Thus, therapeutic strategies that attenuate HSC activation or disrupt fibrogenic signaling pathways may offer dual benefits by limiting fibrosis in MASH while simultaneously improving metabolic homeostasis.

### 3.4 Pancreatic β-Cell dysfunction and the liver-pancreas axis

The liver-pancreas axis refers to the bidirectional communication between hepatocytes and pancreatic β-cells that maintains systemic glucose and lipid homeostasis (56). Under physiological conditions, the liver integrates nutrient and hormonal signals to regulate glycogen storage, gluconeogenesis, and lipid metabolism, while β-cells adjust insulin and glucagon secretion to preserve euglycemia (57).

This regulatory network becomes disrupted in MASH and T2DM. Hepatic insulin resistance diminishes the ability of insulin to suppress gluconeogenesis and promotes *de novo* lipogenesis, driving lipid accumulation and pro-inflammatory signaling in hepatocytes (58). To counter systemic insulin resistance, β-cells increase insulin secretion; however, sustained hyperinsulinemia induces endoplasmic reticulum (ER) stress, β-cell dysfunction, and apoptosis, contributing to progressive deterioration of glucose tolerance (59).

Hepatic inflammation and fibrosis further aggravate systemic insulin resistance through the release of hepatokines such as fetuin-A and FGF21, which impair insulin signaling in peripheral tissues (60). In addition, dysregulation of hepatokines, such as follistatin (FST) and Selenoprotein P (SelP), promotes the progression of MASH and further exacerbates insulin resistance (61–63). Concurrent adipose tissue dysfunction and alterations in gut microbiota composition amplify these metabolic perturbations, reinforcing the pathological loop that links MASH and T2DM (42).

Given this mechanistic interdependence, the liver-pancreas axis represents an important therapeutic target. Strategies that improve hepatic insulin sensitivity, reduce inflammatory activity, and limit fibrogenesis can restore β-cell function and enhance glycemic control. Conversely, therapies aimed at improving β-cell performance or insulin action may mitigate hepatic lipid overload and inflammation (64). This reciprocal relationship provides the basis for developing dual-mechanism interventions capable of treating both MASH and T2DM simultaneously.

### 3.5 Shared miRNAs and lncRNAs involved in the pathogenesis of MASH and T2DM

**Figure 6:**
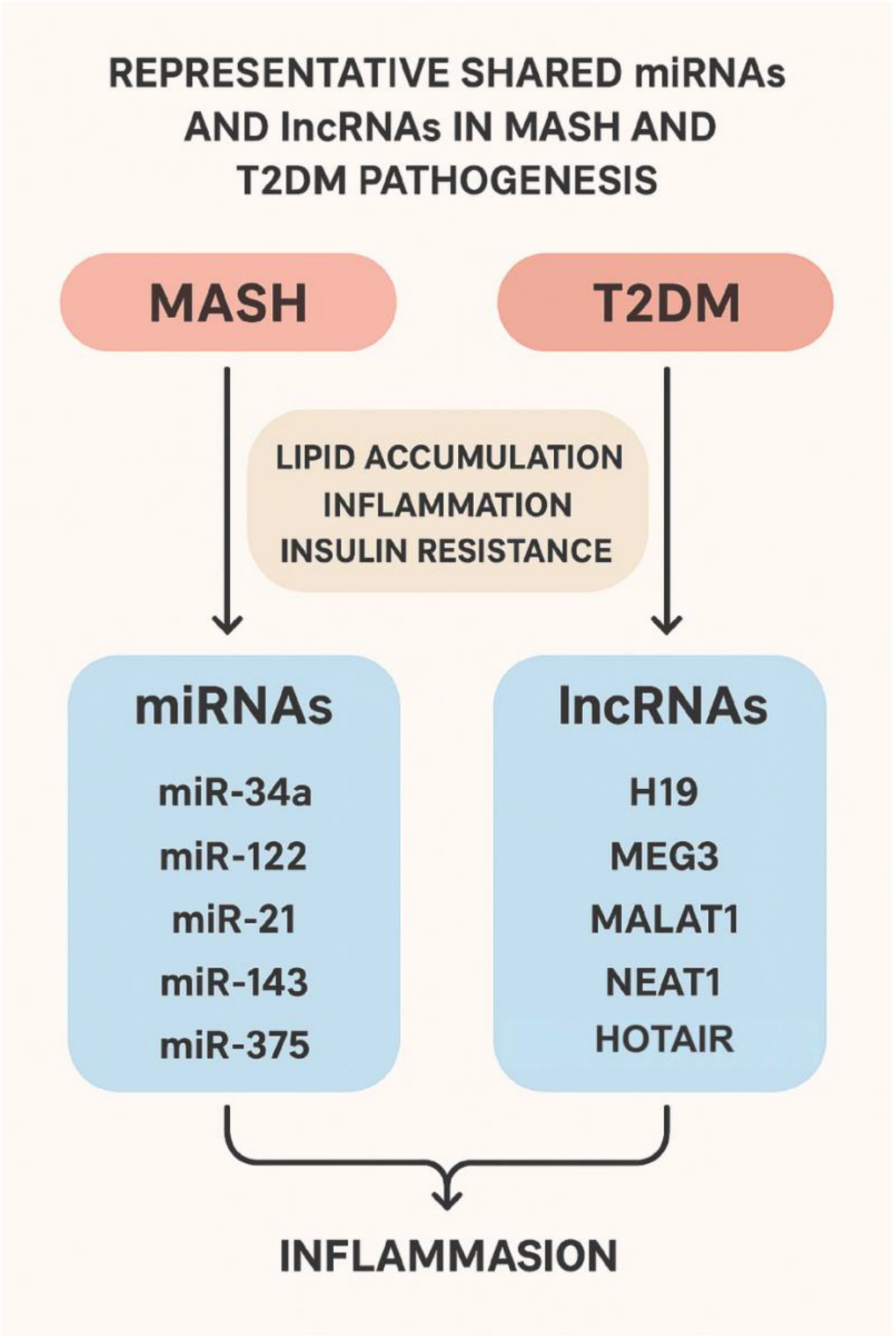
Representative shared miRNAs and lncRNAs involved in the pathogenesis of MASH and T2DM. The schematic summarizes key non-coding RNAs that contribute to overlapping pathogenic mechanisms in Metabolic Dysfunction-Associated Steatohepatitis (MASH) and Type 2 Diabetes Mellitus (T2DM). Central metabolic processes common to both diseases—lipid accumulation, inflammation, and insulin resistance—are shown in the middle. miRNAs (left panel), including *miR-34a, miR-122, miR-21, miR-143*, and *miR-375*, and lncRNAs (right panel), including *H19, MEG3, MALAT1, NEAT1*, and *HOTAIR*, regulate these shared pathways through effects on hepatocyte lipid metabolism, inflammatory signaling, and β-cell/insulin responsiveness. The convergence of these non-coding RNAs highlights their potential roles as mechanistic mediators and biomarkers linking liver and metabolic dysfunction (Created with BioRender.com).

In addition to mRNAs and pathways regulating lipogenesis in hepatocytes, inflammation in Kupffer cells, and activation of hepatic stellate cells during fibrogenesis, non-coding RNAs, such as microRNAs (miRNAs) and long non-coding RNAs (lncRNAs), also play critical roles in the pathogenesis of both MASH and T2DM (**Figure 6**). In this review, we summarize the shared miRNAs and lncRNAs implicated in both conditions.

miR-34a is involved in lipid absorption, inflammation, fatty acid oxidation, and apoptosis. It is overexpressed in both MASH and T2DM patients and participates in a regulatory loop influencing energy and cholesterol homeostasis (65, 66). miR-122 is a liver-specific miRNA that regulates lipid metabolism. Its levels are associated with liver injury and metabolic disorders and are linked to T2DM-related cardiovascular complications (67, 68). miR-21 promotes insulin secretion in islet β cells (69) and activates hepatic stellate cells, contributing to fibrogenesis (70). miR-143 inhibition protects against insulin resistance (71), while elevated miR-143 may serve as a diagnostic biomarker for MASH (72). miR-375 is essential for maintaining normal pancreatic alpha- and beta-cell mass and thus normal glucose homeostasis (73). Its levels are directly correlated with free fatty acids and adipose tissue, serving as a risk marker for MASH (74).

In addition to miRNAs, lncRNAs also participate in the pathogenesis of MASH and T2DM. H19 contributes to both MASH and T2DM by promoting hepatic lipogenesis (75) and enhancing gluconeogenesis while impairing insulin sensitivity (76). HOTAIR has been associated with both T2DM and MASH, with circulating HOTAIR serving as a predictive marker for these conditions (77, 78). MALAT1 increases insulin resistance, and its inhibition improves T2DM (79). Additionally, MALAT1 promotes hepatic fibrosis, highlighting its role in MASH pathogenesis (80). NEAT1 is significantly upregulated in T2DM and influences the development, progression, and prognosis of both MAFLD and T2DM (81). MEG3 upregulation in T2DM contributes to disease prognosis (82), and has been identified as a potential biomarker for MAFLD through its regulation of hepatic lipogenesis (83).

## 4 Preclinical insights into the mechanistic interplay of MASH and T2DM

**Figure 7:**
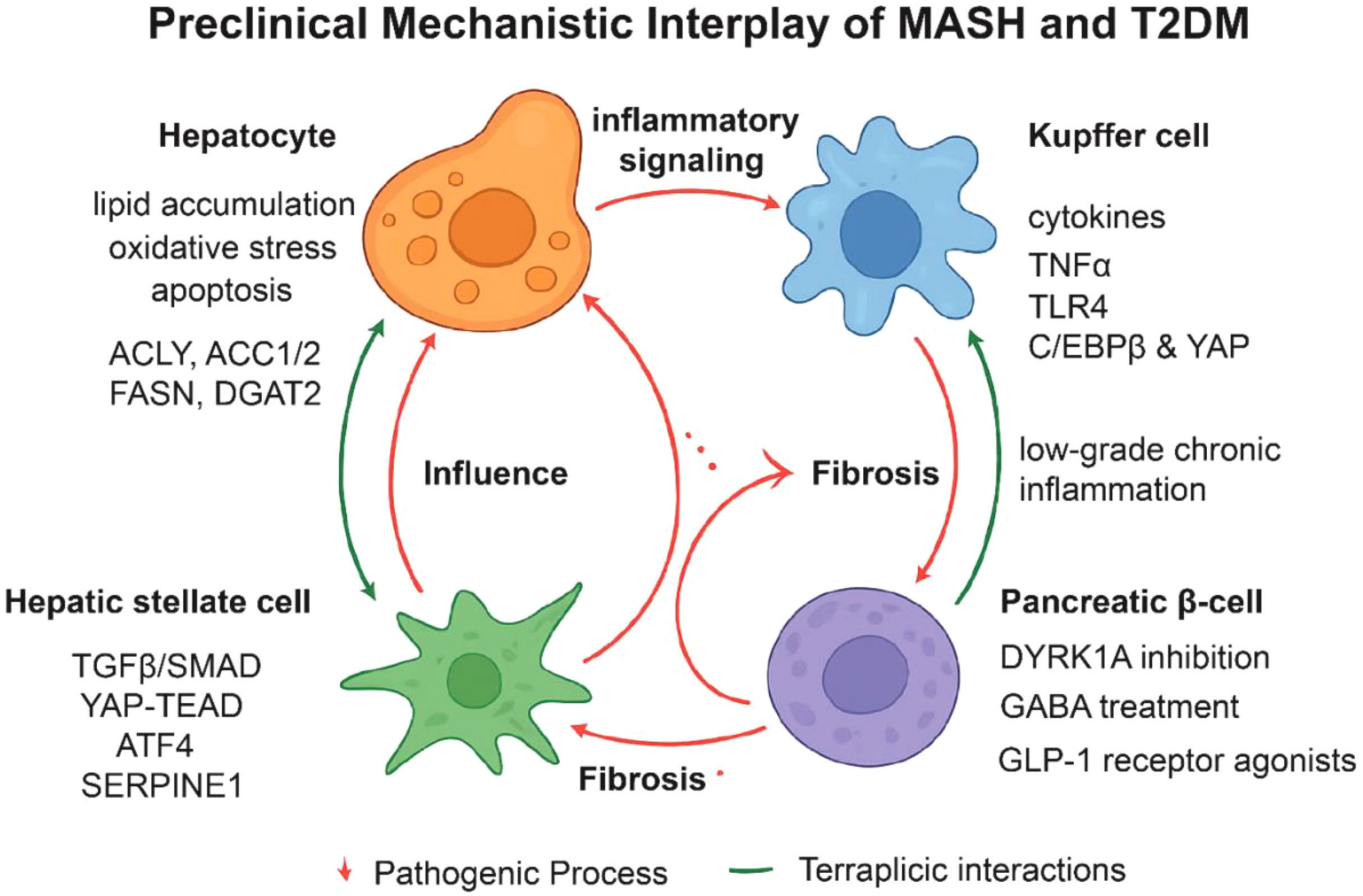
Mechanistic interplay between metabolic dysfunction-associated steatohepatitis (MASH) and type 2 diabetes mellitus (T2DM). The schematic summarizes preclinical cellular and molecular interactions linking hepatocytes, Kupffer cells, hepatic stellate cells (HSCs), and pancreatic β-cells in the progression of MASH and T2DM. Hepatocyte lipid accumulation, oxidative stress, and apoptosis promote inflammatory signaling and contribute to HSC activation. Metabolic pathways implicated in hepatocyte dysfunction include ACLY, ACC1/2, FASN, and DGAT2, with increased ApoB production contributing to disease progression. Kupffer cells secrete cytokines and engage TNFα and TLR4 signaling, modulated by C/EBPβ and YAP activity. Activated HSCs drive fibrosis through TGF-β/SMAD, YAP-TEAD, ATF4, and SERPINE1 pathways. Pancreatic β-cell dysfunction is influenced by impaired insulin secretion and loss of regenerative capacity, with potential interventions including DYRK1A inhibition, GABA treatment, and GLP-1 receptor agonists. Red arrows indicate pathogenic processes contributing to fibrosis and metabolic deterioration, while green arrows denote therapeutic intervention points (Created with BioRender.com).

Preclinical mouse models have been instrumental in elucidating the molecular mechanisms underlying MASH and T2DM and in testing emerging therapeutic strategies. These models allow precise genetic manipulation, dietary interventions, and pharmacological studies in a controlled environment, highlighting four primary cellular targets: hepatocytes, Kupffer cells, hepatic stellate cells (HSCs), and pancreatic β cells, which collectively drive lipid accumulation, inflammation, fibrosis, and insulin resistance (**Figure 7**).

### 4.1 Hepatocyte-targeted interventions

Hepatocytes serve as the central hub of lipid accumulation and metabolic dysregulation in MASH (84). Strategies that inhibit *de novo* lipogenesis (DNL) through modulation of ACLY, ACC1/2, FASN, or DGAT2 effectively reduce hepatic triglyceride content, oxidative stress, and downstream inflammatory signaling (85). ACLY inhibition decreases acetyl-CoA availability and suppresses SREBP-mediated lipogenic gene expression, while ACC inhibition limits malonyl-CoA production and enhances mitochondrial β-oxidation (86). FASN blockade reduces palmitate synthesis and attenuates hepatocyte apoptosis (87), and DGAT2 inhibition prevents excessive triglyceride formation, reducing lipotoxicity (88). Beyond DNL, enhancing lipid export via apolipoprotein B (ApoB) pathways or promoting β-oxidation through PPARα agonists further alleviates hepatocyte stress and mitigates Kupffer cell and HSC activation (89, 90).

### 4.2 Kupffer cell-targeted interventions

Kupffer cells orchestrate hepatic inflammation and contribute to systemic insulin resistance (91). Targeting pro-inflammatory cytokines, such as TNF-α and IL-1α/β, reduces monocyte recruitment, attenuates cytokine signaling, and limits MASH progression (92, 93). Transcriptional regulators C/EBPβ and YAP modulate inflammatory gene expression in Kupffer cells; their deletion diminishes lobular inflammation and fibrogenic signaling (94). Toll-like receptor 4 (TLR4) mediates endotoxin and DAMP-induced NF-κB activation, and Kupffer cell–specific TLR4 inhibition decreases hepatic inflammation and improves insulin sensitivity (95). Chronic Kupffer cell activation also generates reactive oxygen species (ROS), which exacerbate hepatocyte injury and HSC activation; interventions targeting oxidative stress via NADPH oxidase inhibition, Nrf2 activation, or macrophage metabolic reprogramming ameliorate both fibrosis and systemic glucose intolerance (96).

### 4.3 HSC-targeted interventions

HSCs are the principal fibrogenic cells driving ECM deposition and fibrosis in MASH (97). Key pathways regulating HSC activation include TGF-β/SMAD (98), YAP–TEAD (99), RXRα (52), ATF4 (53), and SERPINE1 (100). TEAD1, downstream of YAP, promotes HSC proliferation and fibrogenesis; its inhibition reduces collagen deposition and indirectly diminishes hepatic inflammation (101). RXRα agonists maintain HSC quiescence while modulating hepatocyte lipid metabolism, providing synergistic benefits (52). ATF4 drives fibrogenic gene expression under ER stress, and its inhibition decreases ECM synthesis while improving hepatocyte metabolic function (53). SERPINE1 limits ECM degradation; HSC-specific deletion enhances matrix turnover and reduces fibrosis (100). Collectively, these studies highlight the potential of targeting HSCs to disrupt fibrosis while indirectly mitigating hepatocyte and Kupffer cell-driven pathology.

### 4.4 Pancreatic β cells-targeted interventions

Pancreatic β-cells are essential for maintaining glucose homeostasis, and their dysfunction or loss is a key contributor to T2DM (102, 103). Therapeutic strategies aim to β-cells in T2DM focus on preserving β-cell mass, enhancing functional capacity, or promoting regeneration (104, 105). Approaches that stimulate β-cell proliferation, protect against apoptosis, or induce regenerative processes can improve β-cell function and support glucose regulation. Dual-specificity tyrosine-regulated kinase 1A (DYRK1A) has been identified as a critical regulator of human β-cell proliferation, and inhibition of DYRK1A restores the β-cell mass and function in preclinical T2DM models, improving glucose homeostasis (106, 107). Additionally, γ-aminobutyric acid (GABA) administration has been shown to expand β-cell mass through regenerative effects in diabetic mouse models (108, 109). Glucagon-like peptide-1 (GLP-1) receptor agonists further protect β-cells from apoptosis induced by saturated free fatty acids (FFAs), thereby contributing to T2DM amelioration (110–112).

### 4.5 Combination and multi-target strategies

Single-target interventions frequently yield partial therapeutic effects, whereas combinatorial approaches targeting hepatocytes, Kupffer cells, and HSCs demonstrate superior efficacy. For example, co-inhibition of ACC in hepatocytes and TLR4 in Kupffer cells markedly reduces both steatosis and fibrosis (95, 113). Similarly, combining FASN inhibition with modulation of ATF4 or TEAD1 effectively suppresses MASH progression (101).

Simultaneous targeting of hepatocytes, Kupffer cells, hepatic stellate cells, and pancreatic β-cells offers a synergistic approach to treat both MASH and T2DM by addressing steatosis, inflammation, fibrosis, and insulin resistance. Preclinical studies support this strategy, including evidence that dual inhibition of hepatocyte DGAT2 and stellate cell FASN effectively reduces MASH pathology (114), and reviews highlighting the role of the immune-stellate cell axis in fibrogenesis (115). Additionally, emerging data indicate crosstalk between the liver and β-cells mediated by hepatokines and extracellular vesicles (56). Together, these findings emphasize a multi-target therapeutic paradigm, particularly relevant for advanced or multifactorial metabolic disease.

### 4.6 Translational considerations

While mouse models provide essential mechanistic insights, species-specific differences in lipid metabolism and inflammatory responses must be considered. Nevertheless, preclinical studies have informed the development of therapeutics, including resmetirom (116), semaglutide (117), firsocostat (118), and DGAT2 inhibitors (119), which are currently being evaluated in clinical trials. Timing and disease stage are critical: interventions during early steatosis or mild inflammation are more likely to prevent fibrosis, whereas advanced disease may require combination therapies targeting multiple cell types and signaling pathways (120). These insights underscore the need for personalized treatment strategies tailored to disease stage, metabolic profile, and comorbidities.

## 5 Clinical therapeutic approaches in MASH and T2DM

**Figure 8:**
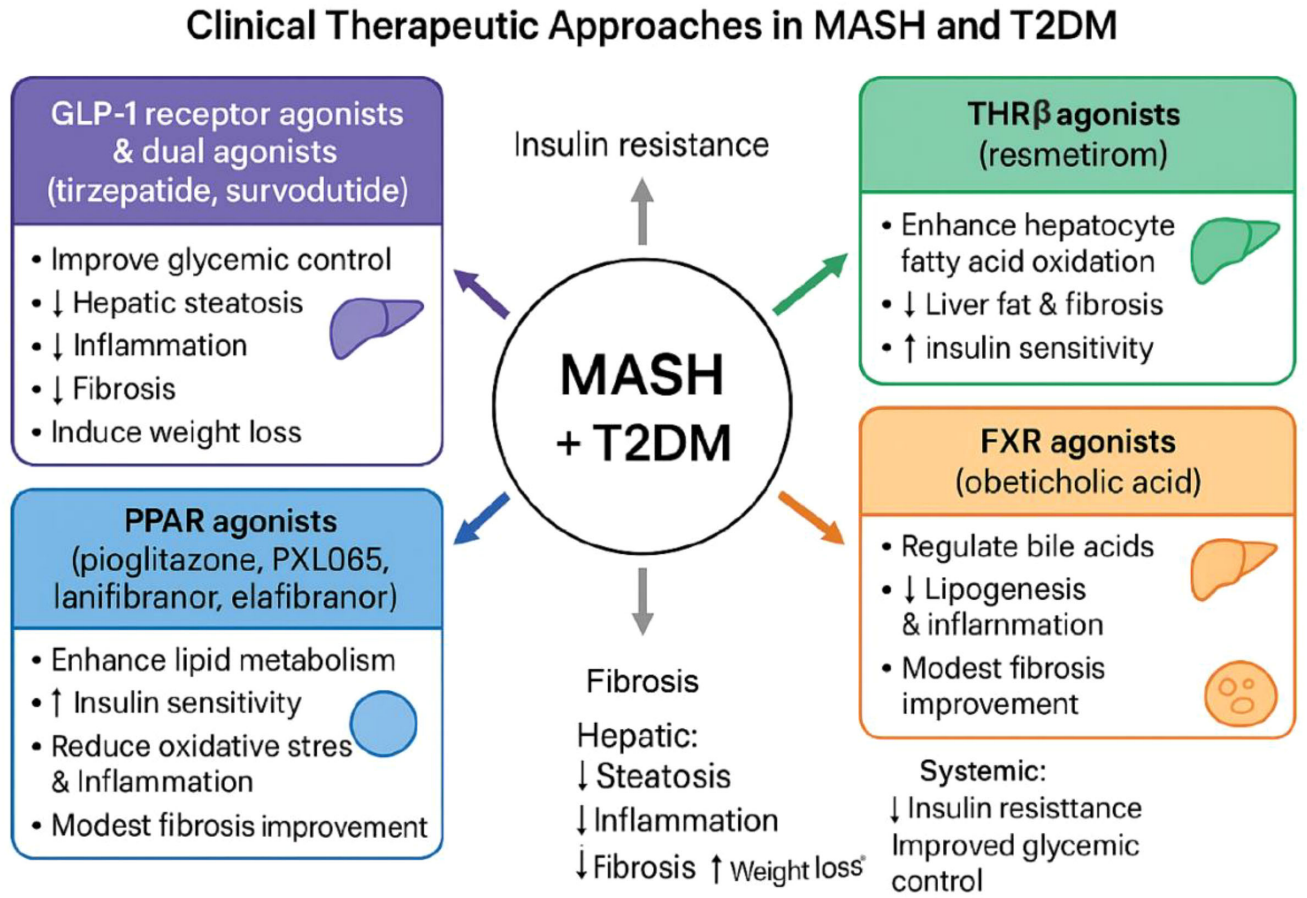
Clinical therapeutic approaches for metabolic dysfunction-associated steatohepatitis (MASH) in the context of type 2 diabetes mellitus (T2DM). The diagram summarizes key pharmacologic classes used or under investigation for the treatment of MASH in individuals with T2DM. GLP-1 receptor agonists and dual agonists (tirzepatide, survodutide) improve glycemic control and reduce hepatic steatosis, inflammation, and fibrosis while promoting weight loss. PPAR agonists (pioglitazone, PXL065, lanifibranor, elafibranor) enhance lipid metabolism, improve insulin sensitivity, and decrease oxidative stress and inflammation, with modest antifibrotic effects. THRβ agonists (resmetirom) increase hepatocyte fatty acid oxidation and reduce liver fat and fibrosis while improving insulin sensitivity. FXR agonists (obeticholic acid) modulate bile acid signaling, reduce lipogenesis and inflammation, and provide modest fibrosis improvement. Collectively, these therapeutic classes target central metabolic and hepatic pathways implicated in steatosis, inflammation, fibrosis progression, and systemic insulin resistance (Created with BioRender.com).

The management of metabolic dysfunction-associated steatohepatitis (MASH) complicated by type 2 diabetes mellitus (T2DM) requires multi-pathway therapeutic strategies due to the systemic and interconnected nature of these conditions (121). Both diseases share overlapping pathogenic mechanisms, including insulin resistance, hepatic lipid accumulation, chronic inflammation, and fibrosis, with bidirectional communication through the liver-pancreas axis (9). Translating preclinical insights into effective clinical therapies has thus emphasized interventions that simultaneously target hepatic pathology and systemic metabolic dysfunction. Over the past decade, several pharmacological classes have emerged as front-runners, including glucagon-like peptide-1 (GLP-1) receptor agonists, dual GLP-1/GIP receptor agonists, peroxisome proliferator-activated receptor (PPAR) agonists, thyroid hormone receptor-beta (THRβ) agonists, farnesoid X receptor (FXR) agonists, and fibroblast growth factor (FGF) analogues (**Figure 8**).

GLP-1 receptor agonists were initially developed to improve glycemic control in T2DM by enhancing insulin secretion, suppressing glucagon, slowing gastric emptying, and promoting satiety (122). Clinical trials have demonstrated that these agents also confer significant hepatic benefits, including reductions in steatosis, inflammation, and fibrosis. Semaglutide, a long-acting GLP-1 receptor agonist approved for non-cirrhotic MASH with moderate to advanced fibrosis, achieved MASH resolution in ~32% more patients versus placebo (123), improved combined steatohepatitis and fibrosis outcomes in ~16.6% of patients, induced substantial weight loss (~8.7%), and improved glycemic parameters (117). Liraglutide, administered daily via subcutaneous injection, shows comparable dual efficacy, increasing MASH resolution by ~30%, reducing fibrosis progression by ~27%, and improving glycemic outcomes, particularly in overweight or obese patients (124). Next-generation dual receptor agonists, such as tirzepatide (GLP-1/GIP) and survodutide (GLP-1/glucagon), further enhance therapeutic potential. Tirzepatide demonstrated 52% MASH resolution and a 21% improvement in one-stage fibrosis at 15 mg, accompanied by significant weight loss (12-15%) and reductions in HbA1c and fasting glucose (125). Survodutide similarly improved MASH by 48%, with ~14% more patients achieving at least one-stage fibrosis improvement (126). Gastrointestinal side effects are the most common for GLP-1–based therapies but are generally mild and manageable (127). These findings underscore the value of GLP-1–based dual and multi-receptor agonists in integrated management of MASH and T2DM.

PPAR agonists, which target lipid metabolism, insulin sensitivity, and inflammation, provide complementary benefits (128). Pioglitazone, a PPARγ agonist, has been shown to improve hepatic steatosis and glycemic control, leading to MASH resolution in approximately 20-30% more patients compared with placebo, although its impact on fibrosis remains modest. Common adverse effects include weight gain and fluid retention (129). PXL065, a deuterium-stabilized derivative of pioglitazone, offers enhanced metabolic benefits with fewer side effects, improving MAFLD activity score in about 20% of patients without worsening fibrosis and reducing the risk of progression to T2DM in susceptible individuals (130). Lanifibranor, a pan-PPAR agonist, achieved MASH resolution without fibrosis worsening in 27% of patients at a 1200 mg dose, and one-stage fibrosis improvement in 26%, along with improvements in insulin sensitivity and fasting glucose levels (131). Elafibranor, a dual PPARα/γ agonist, showed modest efficacy with minimal adverse effects (132). Mechanistically, PPAR agonists promote hepatic lipid processing, reduce oxidative stress, attenuate inflammatory pathways, and enhance systemic insulin sensitivity, highlighting their dual therapeutic potential (133).

Thyroid hormone receptor-beta agonists, exemplified by resmetirom (MGL-3196, Rezdiffra), selectively target hepatocyte metabolism to enhance mitochondrial fatty acid β-oxidation, promote hepatic lipid clearance, and reduce fibrogenesis (116, 134). Phase 2 studies demonstrated 22.5-28.8% reductions in liver fat, while Phase 3 trials reported a 20.2% higher rate of MASH resolution without fibrosis progression (135). Resmetirom also improves systemic insulin sensitivity and lipid profiles, with generally mild gastrointestinal adverse effects (135, 136). By directly modulating hepatocyte energy metabolism, THRβ agonists indirectly attenuate inflammatory and fibrotic pathways, offering a hepatocyte-centered strategy for MASH-T2DM management.

Farnesoid X receptor agonists, such as obeticholic acid, regulate bile acid metabolism, lipid homeostasis, and hepatic inflammation (137). Phase 3 trials demonstrated that 12.8% more patients achieved at least one-stage fibrosis improvement, while MASH resolution improved by ~3% compared with placebo (137). Obeticholic acid also enhanced insulin sensitivity by 24.5%, highlighting its systemic metabolic effects (138). FXR activation reduces hepatic lipogenesis, inhibits hepatic stellate cell activation, and improves glucose metabolism (139). Pruritus is the most common adverse effect but is generally manageable. These mechanisms position FXR agonists as an important therapeutic option targeting both hepatic and systemic pathways in MASH-T2DM comorbidity.

Fibroblast growth factor analogues, including FGF19 and FGF21 derivatives, act as multi-target therapies addressing metabolic, inflammatory, and fibrotic pathways (140). Efruxifermin (FGF21 analogue) reduces hepatic fat fraction by 12-14%, improves fibrosis in 51% of patients, and enhances insulin sensitivity and glycemic control (141). Pegozafermin, a glycopegylated FGF21 analogue, achieves 15-20% fibrosis improvement and 21-35% higher MASH resolution, while also improving systemic metabolic parameters (142). Aldafermin, an FGF19 analogue, contributes to fibrosis improvement and hepatic lipid regulation, although its efficacy is generally less robust than FGF21 analogues (143). FGF-based therapies exemplify multi-target pharmacology capable of simultaneously improving hepatic steatosis, reducing inflammation, reversing fibrosis, and enhancing systemic insulin sensitivity, particularly when used in combination with GLP-1 receptor agonists.

Collectively, clinical trial evidence underscores that targeting interconnected pathways such as insulin resistance, hepatic lipid accumulation, inflammation, and fibrogenesis provides dual benefits in MASH and T2DM (10). Patient stratification based on fibrosis stage, metabolic phenotype, and comorbidities is essential to optimize therapeutic outcomes. Emerging trends include multi-target single agents (tirzepatide, lanifibranor, FGF21 analogues), precision medicine approaches, combination therapies, and biomarker-guided treatment (144). The overarching goal is to simultaneously resolve hepatic pathology and improve systemic metabolic health, ultimately reducing the risk of cirrhosis, liver failure, cardiovascular disease, and T2DM-related complications.

## 6 Conclusion and future perspectives

Type 2 diabetes mellitus and metabolic dysfunction-associated steatohepatitis are tightly linked conditions, with each disease influencing the onset and progression of the other (145). This mutual interaction can accelerate disease severity, increasing patient morbidity and mortality, as well as the burden on healthcare systems (56). The complexity of these disorders arises from overlapping metabolic, inflammatory, and fibrotic pathways, combined with individual differences in genetics and lifestyle, which complicates clinical management (146). Additional challenges include the limited translatability of preclinical models, potential adverse effects such as gastrointestinal disturbance and weight gain, long-term organ toxicity, and restricted access to care, all of which hinder optimal therapeutic implementation (147).

Recent advances have focused on multi-targeted pharmacological interventions capable of simultaneously modulating hepatic lipid metabolism, inflammation, fibrogenesis, and insulin sensitivity (148). Agents such as GLP-1 receptor agonists, dual incretin therapies, PPAR agonists, THRβ modulators, and FGF analogues, in combination with lifestyle modifications, have demonstrated significant improvements in hepatic and metabolic outcomes (149). Looking forward, precision medicine approaches integrating genomic, epigenetic, and microbiome profiling, alongside artificial intelligence and multi-omics analyses, offer opportunities to tailor therapy, predict individual responses, and optimize efficacy while minimizing adverse effects. Non-invasive monitoring and AI-driven optimization may further enhance long-term, real-world management.

Ultimately, addressing MASH and T2DM as interconnected syndromes rather than isolated conditions is critical for developing next-generation, stage-specific, and patient-centered therapies. Integrative strategies that combine pharmacologic innovation, personalized medicine, and lifestyle interventions hold promise not only for reversing hepatic pathology and restoring metabolic homeostasis but also for reducing the global burden of these prevalent chronic disorders. The convergence of mechanistic insights, clinical innovation, and technological advancement positions the field to achieve transformative improvements in patient care.
